# Minor Role of Mitochondrial Respiration for Fatty-Acid Induced Insulin Secretion

**DOI:** 10.3390/ijms140918989

**Published:** 2013-09-16

**Authors:** Nadja Schulz, Oliver Kluth, Martin Jastroch, Annette Schürmann

**Affiliations:** 1 Department of Experimental Diabetology, German Institute of Human Nutrition Potsdam-Rehbruecke, Nuthetal 14558, Germany; E-Mails: nadja.schulz@dife.de (N.S.); oliver.kluth@dife.de (O.K.); 2 Institute for Diabetes and Obesity, Helmholtz Centre Munich, Munich 85764, Germany; E-Mail: martin.jastroch@helmholtz-muenchen.de

**Keywords:** pancreatic islets, insulin secretion, respiratory activity, coupling efficiency

## Abstract

An appropriate insulin secretion by pancreatic beta-cells is necessary to maintain glucose homeostasis. A rise in plasma glucose leads to increased metabolism and an elevated cytoplasmic ATP/ADP ratio that finally triggers insulin granule exocytosis. In addition to this triggering pathway, one or more amplifying pathways—activated by amino acids or fatty acid—enhance secretion by promoting insulin granule recruitment to, and priming at, the plasma membrane. The aim of this study was to clarify the impact of the mitochondrial respiratory activity on fatty acid-induced insulin secretion that was assessed by an extracellular flux analyzer. Treatment of isolated mouse islets with glucose (20 mM) increased insulin secretion 18-fold and correlated with ATP-synthesizing respiration. Furthermore, oxygen consumption rate (*OCR*) significantly increased by 62% in response to glucose, whereas the addition of palmitate resulted only in a minor increase of *OCR* at both 2.8 mM (11%) and 20 mM glucose (21%). The addition of palmitate showed a pronounced increase of coupling efficiency (*CE*) at 2.8 mM glucose but no further insulin secretion. However, treatment with palmitate at 20 mM glucose increased insulin secretion about 32-fold accompanied by a small increase in *CE*. Thus, fatty acid induced respiration has a minor impact on insulin secretion. Our data clearly demonstrate that fatty acids in contrast to glucose play a minor role for respiration-mediated insulin secretion. In the presence of high glucose, fatty acids contribute partially to amplifying pathways of insulin secretion by further increasing mitochondrial activity in the islets of Langerhans.

## 1. Introduction

Insulin secretion from islets initiated in response to nutrients is the main physiological reaction to maintain normal blood glucose levels and markedly depends on ATP. Glucose, fatty acids and amino acids are the three substrates an organism can use to maintain metabolic homoeostasis and are required for the generation of energy (e.g., as ATP). The prime pathway for the degradation of fatty acids is mitochondrial fatty acid β-oxidation [1,2], a key metabolic pathway for energy homoeostasis in organs such as the liver, heart and skeletal muscle. In general, organs and cells are flexible in the choice of the substrate used for energy production. Under normal conditions, glucose is the preferred substrate for oxidation, whereas under specific conditions, e.g., during fasting, fatty acids and ketone bodies become more important as an alternative energy source. This reciprocal relationship or competition between the oxidation of fatty acids and glucose is also known as the glucose-fatty acid or Randle cycle [3].

Pathophysiological conditions like type 2 diabetes are associated with changes in circulating glucose and lipid levels which influence the function of pancreatic islets and insulin secretion [4,5]. Insulin secretion is regulated via two different pathways, the triggering and the amplifying pathway [6]. The triggering pathway includes conversion of glucose to ATP via glycolysis and the respiratory chain, closure of ATP-dependent K^+^ channels with depolarization of the plasma membrane resulting in an influx of calcium and the release of insulin. Superimposed on this pathway, one or more amplifying pathways are described to enhance secretion by promoting insulin granule recruitment and fusion with the plasma membrane. These include the receptor-mediated generation of cAMP and other signals and metabolic mediators such as NADPH [7]. However, all substrates and mechanisms of the amplifying pathways are incompletely understood. Fatty acids have enormous capacity to amplify glucose-stimulated insulin secretion [8,9] which is particularly operative in situations of beta-cell compensation for insulin resistance. They for instance bind to the free fatty acid receptor 1 resulting in enhancement of glucose-stimulated accumulation of cytosolic Ca^2+^ and consequently insulin secretion [10,11]. However, chronically elevated fatty acids, particularly in the presence of elevated glucose levels, reduce insulin biosynthesis [12], secretion [13] and participate in beta-cell loss [4,14,15].

Since it is known that both the triggering and the amplifying pathways are absolutely dependent on the mitochondrial metabolism of glucose, the aim of this study was to investigate the contribution of palmitate-induced insulin secretion by activation of the respiratory chain. Recently, Wikstrom *et al*. [16] described the development of a novel islet respirometry assay to determine the bioenergetic efficiency of islets as a tool to study islet mitochondrial function. Thus, in parallel to measurements of insulin secretion in isolated islets treated with glucose and palmitate we used the Seahorse XF24 extracellular flux analyzer to evaluate oxygen consumption rate and to calculate ATP synthesis, proton leak and coupling efficiency.

## 2. Results and Discussion

**Figure 1:**
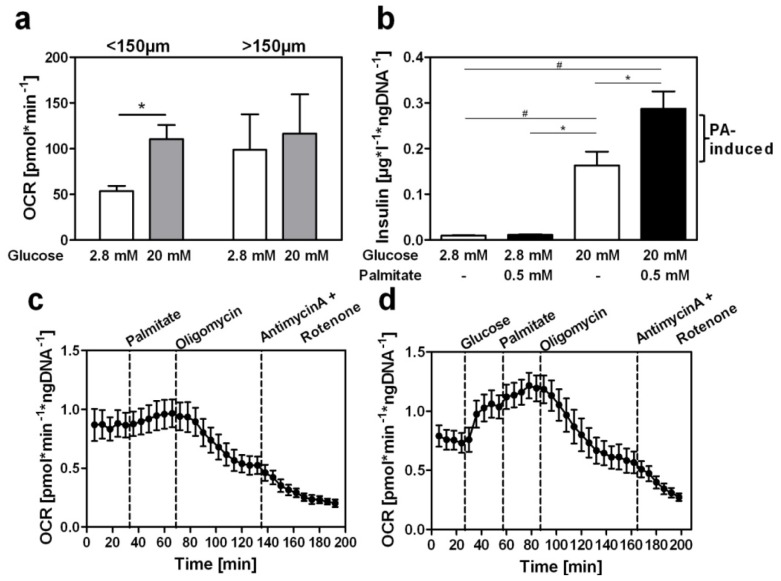
Increased oxygen consumption in islets <150 μm in response to glucose. (**a**) Oxygen consumption rate (*OCR*) of isolated islets <150 μm or >150 μm after glucose stimulation from 2.8 mM to 20 mM; (**b**) Elevated insulin secretion of isolated islets after stimulation with glucose and palmitate. Islets of B6.V-*Lep^ob^* mice were treated with the indicated glucose concentrations in the absence or presence of 0.5 mM palmitate (PA) in Krebs-Ringer buffer for 1 h. Insulin was measured by an ELISA and normalized to islet DNA content; (**c**) Time course of oxygen consumption rate (*OCR*) after application of different stimuli (dashed lines) at a low glucose concentration (2.8 mM); (**d**) Time course of *OCR* during elevation of glucose from 2.8 mM to 20 mM and subsequent addition of further stimuli (dashed lines). Values represent means of 5–10 independent experiments with islets obtained from different mice ± SEM; *p < 0.05; ^#^*p* < 0.01.

Before comparing the capacities of nutrient-stimulated insulin secretion and the mitochondrial metabolism of intact cells the detection of oxygen consumption with the Seahorse XF24 extracellular flux analyzer was optimized. In previous studies islets of both DBA/2J and B6.V-*Lep^ob^* mice were used without showing differences in their glucose-dependent increase in oxygen consumption rate (*OCR*). Since B6.V*-Lep^ob^* mice exhibit a higher number of islets the following experiments were exclusively performed with islets of this mouse strain. We tested the oxygen consumption of islets of different size and observed that islets with a maximal size of 150 μm but not islets larger than 150 μm gave a significant increase of *OCR* in response to elevated glucose concentrations (Figure 1a). In contrast to the *in vivo* situation isolated islets are not connected to vessels and consequently a limited diffusion of oxygen from the inner beta cells of larger islets and/or an impaired fuel supply may occur. Therefore all following studies were performed with islets <150 μm.

Figure 1b illustrates the insulin secretion of isolated islets that were exposed to both low and high glucose concentrations in either the absence or presence of palmitate. The basal release of insulin at a glucose concentration of 2.8 mM increased 1.2-fold in response to 0.5 mM palmitate. Under high glucose condition (20 mM) insulin secretion increased 18-fold and the addition of 0.5 mM palmitate resulted in a further augmentation of insulin release through fatty-acid stimulation (32-fold in comparison to 2.8 mM glucose without palmitate). These data confirmed that in the acute state fatty acids enhance the glucose-stimulated insulin secretion [14,17,18], thereby strengthening the hypothesis that an activated triggering pathway is essential for a sufficient increase in insulin release via the amplifying pathway [19].

In a parallel set of experiments we used isolated islets to evaluate the impact of palmitate at low (2.8 mM) and high (20 mM) glucose concentrations for the oxygen consumption via mitochondrial metabolism. With the extracellular flux analyzer we observed that 0.5 mM palmitate increased basal *OCR* by 11% (Figure 1c). In contrast, basal *OCR* was increased by 62% in response to 20 mM glucose. The subsequent addition of 0.5 mM palmitate in the presence of 20 mM glucose increased the *OCR* by 21% (Figure 1d), indicating that palmitate is generally metabolized for ATP production via the respiratory chain however, to a low extent in comparison to its strong effect on insulin secretion. Thereby, we have demonstrated for the first time that fatty acids induce a modest activation of mitochondrial metabolism and ATP synthesis in isolated islets and conclude that their ability to augment insulin secretion at high glucose conditions is mainly mediated via the amplifying pathway. The detection of the ATP/ADP ratio of isolated islets treated with glucose alone or glucose plus palmitate would give further information about the mitochondrial capacity. Due to limited islet material this approach could not be included in the actual study, however it will be a focus in future experiments using beta-cell lines such as Min6 cells.

**Figure 2:**
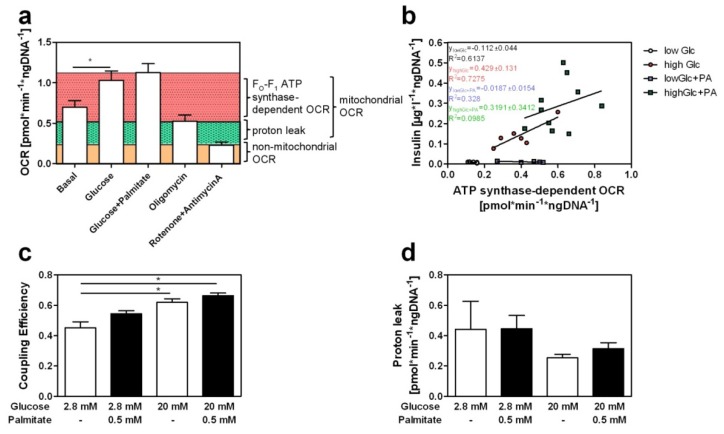
Impact of palmitate on mitochondrial respiration. (**a**) Changes in oxygen consumption rate (*OCR*) of isolated islets in response to indicated stimuli; (**b**) Correlation of insulin secretion and ATP synthase-dependent *OCR* in response to 2.8 mM glucose (low Glc) or 20 mM glucose (high Glc) without or with 0.5 mM palmitate (+PA). In parallel measurements of insulin secretion and oxygen consumption were performed at different conditions with isolated islets. Islets were treated with Krebs-Ringer buffer for 1 h. Insulin was measured by an ELISA and normalized to islet DNA content. ATP synthase-dependent *OCR* was calculated by subtracting mitochondrial proton leak from the respiration under the indicated conditions; (**c**) Calculation of islet coupling efficiency in response to 2.8 mM or 20 mM glucose without and with 0.5 mM palmitate (caluculated as: *CE* = 1 − (*OCR*_proton leak_/*OCR*_mito.respiration_)); (**d**) Proton leak of isolated islets after treatment with indicated substrates (calculated as: *OCR*_proton leak_ = *OCR*_mito.respiration_ − *OCR*_ATPsynthase_). Values in (**a**), (**c**) and (**d**) represent means of 5–10 independent experiments with islets obtained from different mice ± SEM; plotted points in (**b**) represent secreted insulin of one experimental animal and the corresponding ATP synthase-dependent *OCR* value at a particular condition. *p < 0.05; ^#^*p* < 0.01.

We next measured *OCR* after the application of different stimuli to calculate the coupling efficiency (*CE*) which reflects the efficiency of mitochondrial energy conversion after blocking ATP production and mitochondrial respiration. The basal *OCR* of 0.69 ± 0.08 pmol·min^−1^·ng DNA^−1^ was elevated to 1.03 ± 0.11 pmol·min^−1^·ng DNA^−1^ by 20 mM glucose (Figure 2a). In order to study the relationship between insulin secretion and ATP synthase-dependent *OCR* we calculated linear regression during different stimulatory conditions. Thereby, we were able to determine insulin secretion and *OCR* of isolated islets from the same animal. We calculated a strong correlation between ATP synthase-dependent *OCR* and insulin secretion of isolated islets treated with low glucose (low Glc; *R*^2^ = 0.6137) or high glucose concentrations (high Glc; *R*^2^ = 0.7275). In contrast, no correlation between ATP synthase-dependent *OCR* and insulin secretion exists in response to palmitate either at low glucose (low Glc + PA; *R*^2^ = 0.0328) or at high glucose exposure (high Glc + PA; *R*^2^ = 0.0985) (Figure 2b). Calculations of *CE* which is about 0.43 in the basal state showed a significant increase in response to glucose (to 0.60), whereas palmitate induced only a slight increase of *CE* at low (0.54) and high glucose concentrations (0.66) (Figure 2c). The weak increase of oxygen consumption and coupling efficiency in response to palmitate indicates that the amplification itself is nearly independent of mitochondrial activity. However, since we cannot exclude non-linear kinetics of mitochondrial respiration and insulin secretion a parallel detection of the membrane potential of isolated beta-cells will be required in future studies. The amplifying pathways are very poorly understood, in terms of how fatty acids and/or amino acids couple to the mechanics of exocytosis of insulin carrying granules [20,21]. Alquier *et al*. [10] suggested that binding of fatty acids to a G-protein coupled receptor, the free fatty acid receptor 1, results in elevation of intracellular calcium as a part of the amplifying pathway [10,11]. Moreover, fatty acids are known to be degraded in mitochondria via beta-oxidation. However, high concentrations of glucose and its metabolite malonyl-CoA inhibit carnitine palmitoyltransferase 1 (CPT1) and the subsequent import of fatty acids into the mitochondria [20,21] leading to an accumulation of fatty acids in the cytosol. This overwhelming increase of fatty acids within the cell has two consequences, on the one hand the production of toxic ceramides [21] and on the other hand the ATP-consuming storage of lipids as triglycerides [5,20]. This cycling of lipids and its intermediates after fatty-acid stimulation is suggested to produce signals which participate in the amplifying pathway and augments insulin secretion nearly independent of fatty-acid induced mitochondrial activation.

Furthermore, calculations of proton leak confirmed that islets are highly uncoupled [16]. However, no alterations in uncoupling resulted after stimulation with glucose or palmitate (Figure 2d). Fatty acids are described to produce superoxides [22,23] and to activate uncoupling via UCP2, a highly expressed proton transporter in islets of Langerhans [23,24]. During the process of uncoupling protons of the respiratory chain reenter the mitochondrial matrix through a so called proton leak mediated by e.g., UCP2 without producing ATP. In contrast to former studies performed with isolated mitochondria of INS1 cells [25,26] we showed that fatty acids did not have an impact on proton leak when applied to isolated islets. This might be due to the fact that the characterization of mitochondrial action of the entire islet has the disadvantage that not all cells are beta-cells and that the other cell types (e.g., alpha-cells) also participate in oxygen consumption. Therefore, further studies with isolated and FACS sorted beta-cells are needed to specify their exact contribution. These data indicate that a sufficient defense of isolated islets exists against fatty-acid mediated reactive oxygen species production under short-term stimulation. Additionally, palmitate is probably not involved in the regulation of uncoupling in isolated mammalian islets of Langerhans. However, these data should be confirmed by studies of beta-cells lines such as INS cells with regard to short- and long-term incubation of fatty acids. It would be possible that fatty acids increase proton leak due to prolonged exposure when defense against reactive oxygen species becomes insufficient and thus insulin secretion would be influenced by superoxide production and uncoupling.

In summary, we showed that fatty acids exhibit a low capacity to increase respiratory activity in isolated islets especially at low glucose concentrations when palmitate has no apparent influence on insulin secretion. Moreover, palmitate appears to play only a minor role in fatty-acid activated mitochondrial respiration under high glucose conditions and cannot explain the 32-fold enhancement of insulin secretion that was induced by the addition of palmitate at 20 mM glucose. The minor role of fatty acids for the induction of insulin secretion via an increased mitochondrial response and ATP production is plausible. If a general increase of fatty acids—for instance in response to fasting when blood glucose levels are low and free fatty acids levels increase—would result in an elevated beta-oxidation and thereby in an increased ATP production, its induction of insulin release should decrease the blood glucose levels to a dramatically low level. Therefore, the inferred role of malonyl-CoA is that it is essential for maintaining glucose homeostasis because it derives from glucose metabolism [27] and inhibits fatty acid oxidation by allosteric inhibition of CPT1. This increases the availability of LC-CoA for lipid signaling to cellular processes that are needed to increase exocytosis of insulin granuels.

## 3. Experimental Section

### 3.1. Mice

Studies of islet-size dependent function were performed with islets from 24 week old male DBA/2J. All following analyses were carried out with isolated islets form 22–26 week old male B6.V-*Lep^ob^* mice. Animals were housed in air conditioned rooms (temperature 20 ± 2 °C) with a 12 h light–dark cycle in accordance with the National Institutes of Health guidelines for the care and use of laboratory animals. All experiments were approved by the ethics committee of the State Agency of Environment, Health and Consumer Protection (State of Brandenburg, Germany).

### 3.2. Isolation of Islets of Langerhans

Pancreatic islets were isolated by injection of collagenase-P (Roche, Mannheim, Germany), in the common bile duct as described [17]. Isolated islets recovered for 24–48 h in 1640-RPMI (PAA, Laborbedarf, Austria) containing 11 mmol/L glucose, 10% FCS gold, 100 U/mL penicillin, 100 U/mL streptomycin in humidified 5% CO_2_, 95% air at 37 °C. Islets were separated under the microscope (Leica DFC420, Wetzlar, Germany) as <150 μm or >150 μm to study changes in response to stimuli with regard to islet size.

### 3.3. Preparation of BSA-Conjugated Palmitate

Palmitate has a low solubility in aqueous solutions. To produce an aqueous-soluble and absorbable fatty acid complex we conjugated palmitate (Sigma, St. Louis, MO, USA) with bovine serum albumin (BSA, essentially fatty acid free; Sigma). Preparation of BSA-conjugated palmitate was performed as described previously [28]. Briefly, sodium palmitate solution preheated to 75 °C was slowly dissolved in a 48 °C preheated BSA solution. Palmitate was complexed in a 6 to 1 molar ratio with BSA. To exclude possible side effects of BSA we compared islets of Langerhans treated with BSA-conjugated palmitate and BSA alone (Figure S1).

### 3.4. Detection of Insulin Secretion

Isolated islets were kept in Krebs-Ringer buffer containing 2.8 mM glucose for 1 h and then incubated at 2.8 mM or 20 mM glucose without and with 0.5 mM palmitate (PA) for 1 h. Insulin content of supernatant was measured by Mouse Insulin Ultrasensitive ELISA (DRG Instruments, Germany) and normalized to the DNA content of islets.

### 3.5. Measurement of Oxygen Consumption Rate

Oxygen consumption rates (*OCR*) of isolated islets were determined with an XF24-3 extracellular flux analyzer (Seahorse Bioscience, Billerica, MA, USA). The analyzer uses fluorescence sensors for detection of rate changes of dissolved O_2_ in the surrounding media of isolated islets. To determine the flux of oxygen a chamber of ~7 μL was created mechanically within the multi-well plate during the measurement [29]. After each measurement a period of mixing and waiting followed to re-equilibrate the islets with the whole buffer volume. Four different ports (A, B, C and D) nearby the fluorescence sensor enabled a controlled injection of substances to the media. To measure cellular and mitochondrial oxygen consumption rate 70 islets were placed in 500 μL Krebs-Ringer buffer (111 mM NaCl, 4.7 mM KCl, 2 mM MgSO_4_, 1.2 mM Na_2_HPO_4_, 0.5 mM carnitine, pH 7.4) containing 2.8 mM glucose in an islet capture microplate. Islets were incubated at 37 °C without CO_2_ for 1 h before starting the experiment. Measurement of *OCR* was performed according to manufacture’s instructions, with duration for mixing, waiting and measurement of 1, 2 and 3 min, respectively. The treatment time with each of the compounds was adjusted to reach steady state conditions (plateau phase of *OCR*). After measurement of *OCR* at basal conditions (2.8 mM glucose) different substrates were injected in separated experiments: (a) no further substrate application, to measure at low glucose conditions (low Glc); (b) 0.5 mM palmitate (low Glc + PA); (c) 20 mM glucose (high Glc); (d) 20 mM glucose followed by 0.5 mM palmitate (high Glc + PA). Each condition was followed by an application of oligomycin (6 μM) as penultimate step before a cocktail of rotenone (5 μM) and antimycin A (4 μM) was injected. Using different inhibitors enabled analysis of islet respiration. Oligomycin inhibits F_O_ subunit of ATP synthase and therewith it is suitable to calculate mitochondrial ATP synthase-dependent *OCR*. Additionally, rotenone and antimycin A block mitochondrial electron flux of complex I and III of the respiratory chain whereby determination of non-mitochondrial respiration is possible. Mitochondrial respiration (*OCR*_mito.respiration_) represents the sum of oxygen consumption due to the proton leak (green colored segment Figure 2a) and ATP synthesis-linked respiration (pink colored segment Figure 2a), whereas non-mitochondrial respiration (*OCR*_Rotenone/Antimycin A_) was subtracted. ATP synthase-dependent *OCR* was calculated as followed: *OCR*_ATPsynthase_ = (*OCR*_condition_ − *OCR*_Roteneone/Antimydin A_) − (*OCR*_Oligomycin_ − *OCR*_Rotenone/Antimycin A_). To evaluate proton leak, which describes flux of protons through the mitochondrial membrane thereby producing heat but no ATP, we used the equation: *OCR*_proton leak_ = *OCR*_mito.respiration_ − *OCR*_ATPsynthase_. Moreover, we calculated the efficiency of mitochondrial energy conversion, termed coupling efficiency (*CE*) as the proportion of respiration converted to ATP, considering the proportion that is lost as heat by proton leak (*CE* = 1 − (*OCR*_proton leak_/*OCR*_mito.respiration_)) [26]. For normalization DNA content of islets was measured with Quant-iT PicoGreen (Invitrogen, Darmstadt, Germany).

### 3.6. Statistical Analysis

Data are presented as mean ± SEM. Statistically significant differences between conditions were defined as *p* < 0.05 by using two-tailed Student's *t* test and one way ANOVA.

## 4. Conclusions

In conclusion, glucose-induced insulin secretion of isolated islets is mediated by an increase in mitochondrial respiration and coupling efficiency, known as the triggering pathway. In contrast, a further augmentation of insulin secretion through palmitate at high glucose concentrations is only mediated to a minor extent via mitochondrial oxygen consumption indicating that palmitate induces insulin secretion nearly exclusively through the amplifying pathway.
